# Effect of the L499M mutation of the ascomycetous *Botrytis aclada* laccase on redox potential and catalytic properties

**DOI:** 10.1107/S1399004714020380

**Published:** 2014-10-23

**Authors:** Evgeny Osipov, Konstantin Polyakov, Roman Kittl, Sergey Shleev, Pavel Dorovatovsky, Tamara Tikhonova, Stephan Hann, Roland Ludwig, Vladimir Popov

**Affiliations:** a A. N. Bach Institute of Biochemistry, Leninsky Prospect 33/2, Moscow 119071, Russian Federation; b Engelhardt Institute of Molecular Biology, Vavilova Str. 32, Moscow 119991, Russian Federation; c BOKU – University of Natural Resources and Life Sciences, Muthgasse 18, 1190 Wien, Austria; d RSC ‘Kurchatov Institute’, Acad. Kurchatov Sq. 1, Moscow 123182, Russian Federation; e Biomedical Sciences, Health and Society, Malmö University, 205 06 Malmö, Sweden

**Keywords:** laccase, *Botrytis aclada*, T1-site mutant, redox potential

## Abstract

Laccases are members of a large family of multicopper oxidases that catalyze the oxidation of a wide range of organic and inorganic substrates accompanied by the reduction of dioxygen to water. These enzymes contain four Cu atoms per molecule organized into three sites: T1, T2 and T3. In all laccases, the T1 copper ion is coordinated by two histidines and one cysteine in the equatorial plane and is covered by the side chains of hydrophobic residues in the axial positions. The redox potential of the T1 copper ion influences the enzymatic reaction and is determined by the nature of the axial ligands and the structure of the second coordination sphere. In this work, the laccase from the ascomycete *Botrytis aclada* was studied, which contains conserved Ile491 and nonconserved Leu499 residues in the axial positions. The three-dimensional structures of the wild-type enzyme and the L499M mutant were determined by X-ray crystallography at 1.7 Å resolution. Crystals suitable for X-ray analysis could only be grown after deglycosylation. Both structures did not contain the T2 copper ion. The catalytic properties of the enzyme were characterized and the redox potentials of both enzyme forms were determined: *E*_0_ = 720 and 580 mV for the wild-type enzyme and the mutant, respectively. Since the structures of the wild-type and mutant forms are very similar, the change in the redox potential can be related to the L499M mutation in the T1 site of the enzyme.

## Abstract 2

The structures of the ascomycetous *B. aclada* laccase and its L499M T1-site mutant have been solved at 1.7 Å resolution. The mutant enzyme shows a 140 mV lower redox potential of the type 1 copper and altered kinetic behaviour. The wild type and the mutant have very similar structures, which makes it possible to relate the changes in the redox potential to the L499M mutation

## 1. Introduction   

Laccases (EC 1.10.3.2; benzenediol:oxygen oxidoreductases) belong to the multicopper oxidase family, which also includes ascorbate oxidase and ceruloplasmin (Solomon *et al.*, 1996 ▶). Laccases catalyze the oxidation of a wide variety of substrates accompanied by the reduction of molecular oxygen to water. These enzymes are abundant in nature and are found in plants, fungi, some insects and bacteria, where they are involved in processes as different as lignification, delignification, morphogenesis and biosynthesis. Owing to the broad substrate specificity and the formation of nontoxic products, laccases have attracted interest because of their potential applications in wastewater treatment, delignification of soils, bleaching of paper pulp and decolorization of textile dyes (Xu, 2002 ▶).

The first three-dimensional structure of laccase was determined in 1998 for the enzyme from *Coprinus cinereus* (Ducros *et al.*, 1998 ▶). To date, the structures of 11 bacterial and 15 eukaryotic laccases have been determined, including 13 laccases from basidiomycetes and two from ascomycetes: *Melanocarpus albomyces* (*Ma*L; Hakulinen *et al.*, 2002 ▶) and *Thielavia arenaria* (*Ta*L; Kallio *et al.*, 2011 ▶). The known laccases and ascorbate oxidase have similar three-dimensional structures. They are composed of two or three cupredoxin-like domains. The active site of laccase comprises four Cu atoms. Based on their optical and EPR properties, these atoms are classified as type 1 copper (Cu1) in the T1 site and an ensemble of type 2 and type 3 coppers (Cu2, Cu3_1_ and Cu3_2_) in the trinuclear T2/T3 site (Solomon *et al.*, 1996 ▶). The substrate oxidation takes place at the T1 site and molecular oxygen is reduced to water at the T2/T3 site.

The Cu1 ion is coordinated by the side chains of two histidine residues and one cysteine residue. The copper ion and the atoms involved in its coordination lie in the same plane, on both sides of which are the side chains of the axial hydrophobic residues. The isoleucine residue, which is strictly conserved in all structurally characterized three-domain laccases, is located on one side of this plane, while phenyl­alanine, leucine or methionine residues may be located on the opposite side (Ducros *et al.*, 1998 ▶; Hakulinen *et al.*, 2002 ▶; Enguita *et al.*, 2003 ▶). The Cu1 ion is the primary electron acceptor and its redox potential is responsible for the catalytic efficiency of laccase. The redox potentials of the T1 site of laccases from different organisms vary over a wide range from 0.43 V (*Rhus vernicifera* laccase) to 0.79 V (*Trametes* spp.; Shleev *et al.*, 2005 ▶). For the laccases from *T. villosa* and *Bacillus subtilis*, mutant forms containing all three of the above-mentioned types of residues in the position of the nonconserved axial ligand were obtained, and their spectroscopic and electrochemical properties were studied (Xu *et al.*, 1999 ▶; Durão *et al.*, 2006 ▶). It has been shown that the enzyme containing methionine in the axial position has the lowest redox potential. The replacement of the conserved isoleucine residue by alanine in *B. subtilis* laccase results in a 150 mV decrease in the redox potential. In the structure of this mutant, the Cu1 ion is coordinated by a solvent molecule (Durão *et al.*, 2008 ▶).

The T2/T3 site contains three copper ions (Cu2, Cu3_1_ and Cu3_2_) and eight coordinating histidine residues. The Cu2 ion is coordinated by two histidine residues and in addition can be coordinated by one or two oxygen ligands, resulting in the formation of a square-planar structure. The Cu3_1_ and Cu3_2_ ions are coordinated by six histidine residues and are linked to each other *via* an oxygen ligand. Both T3 copper ions are in a distorted tetrahedral coordination. The Cu2 ion can be selectively removed from laccase by treatment with chelating agents, resulting in the formation of the inactive T2 copper-depleted form of the enzyme (Reinhammar & Oda, 1979 ▶; Koroleva *et al.*, 2001 ▶). The three-dimensional structures of several T2 copper-depleted laccases have been determined (Ducros *et al.*, 1998 ▶, 2001 ▶; De la Mora *et al.*, 2012 ▶).

Of special interest are laccases that are not inhibited by halide ions, particularly by chloride, which is often present at high concentrations in biotechnological processes. A high redox potential of Cu1 broadens the applicability of the enzyme and enables the use of redox mediators exhibiting high oxidation potentials for substrates. The recently found laccase from the ascomycetous fungus *Botrytis aclada* (*Ba*L) combines both beneficial properties: it has a high tolerance for chloride (*I*_50_ = 1.4 *M*) and, as shown in this study, has an unusually high redox potential of the T1 site for an ascomycete laccase (Kittl *et al.*, 2012 ▶).

In the present study, we prepared the L499M mutant of the laccase from *B. aclada*, in which the nonconserved leucine residue in the axial position of the T1 site was replaced by a methionine. The three-dimensional structures of the wild-type and mutant forms of the enzyme were determined, and the kinetic characteristics and redox potentials of both forms were measured.

## 2. Materials and methods   

### 2.1. Gene cloning   

Site-directed mutagenesis was used to create an enzyme variant with an L499M amino-acid exchange. Therefore, a PCR with the overlapping primers 5L499M (5′-CATCTGA­GGGGATGGCAATGC-3′) and 3L499X (5′-CCCTCAGAT­GCGTGCCATGC-3′) was performed with the expression vector p*Ba*Lac based on the pPICZB vector containing the full-length cDNA of *Ba*L used for expression of the wild-type enzyme. Subsequently, the PCR product was isolated and digested with *Dpn*I for 2 h at 37°C to remove any template plasmid (Li & Wilkinson, 1997 ▶). For secreted expression of the laccase cDNA in *Pichia pastoris*, the expression vectors pPICZB and pPICZαA (Invitrogen) were cut with the same restriction enzymes as the amplified fragments (*Pml*I and *Xba*I), ligated with the respective fragment using T4 DNA ligase at 4°C overnight and transformed into chemically competent *Escherichia coli* DHα cells (Invitrogen, Carlsbad, California, USA), and a clone carrying the desired mutation was verified by sequencing. The mutated plasmid p*Ba*LL499M was linearized with *Pme*I at 37°C for 1 h and transformed into *P. pastoris* strain X33. Positive transformants were selected on YPD plates (1% yeast extract, 2% peptone, 0.4% dextrose, 2% agar) containing zeocin (100 µg ml^−1^) as the selective marker and screened on indicator agar plates with BMM agar [100 m*M* potassium phosphate buffer pH 6.0, 3.4 g l^−1^ yeast nitrogen base without amino acids and ammonium sulfate (Amresco, Solon, USA), 1% ammonium sulfate, 400 µg l^−1^ biotin, 0.5% methanol, 2% agar] containing 0.2 m*M* ABTS and 0.1 m*M* CuSO_4_.

### 2.2. Gene expression and protein purification   

*Ba*L was recombinantly expressed in *P. pastoris* and purified as described in Kittl *et al.* (2012 ▶). L499M *Ba*L was expressed and purified in a similar manner. Purification data are summarized in Supplementary Table S1^1^.

The purity of the protein samples was assessed by SDS–PAGE. Mini-PROTEAN TGX precast gels (Bio-Rad) with a gradient from 4 to 15% were used. Protein bands were visualized by staining with Bio-Safe Coomassie, and unstained Precision Plus Protein Standard was used for mass determination. All procedures were performed according to the manufacturer’s recommendations (Bio-Rad).

### 2.3. Circular dichroism   

CD spectra were recorded for *Ba*L and its L499M variant at room temperature in the 195–260 nm wavelength range using a Chiroscan CD spectrophotometer (Applied Photophysics, Leatherhead, England). The measurements were carried out using a 1 mm path-length cell and a protein concentration of 0.125 mg ml^−1^ with a scan rate of 6 nm min^−1^. The sampling wavelength was set to 1 nm and the spectral bandwidth to 3 nm.

### 2.4. Determination of the copper content   

The Cu content was determined using the Cu/S stoichiometry of the investigated proteins following the principle described by Hann *et al.* (2004 ▶). An Ultimate 3000 chromatography system operated using the *Chromeleon* chromatography management system (Thermo Fisher, Sunnyvale, California, USA) equipped with an SEC column (BioSuite 125, 300 µl min^−1^ flow rate, 300 × 4.6 mm, 4 µm particle diameter; Waters, Milford, Massachusetts, USA) was used. The SEC eluent was 100 m*M* ammonium acetate pH 6.5. All chemicals were of ultrapure grade, and ultrapure sub-boiled water was used for preparation of the eluents and dilutions.

An ELAN 6100 DRCII inductively coupled plasma dynamic reaction cell mass spectrometer (ICP-DRC MS; Perkin Elmer SCIEX, Canada) with a PFA nebulizer and a cyclonic spray chamber was combined online with the chromatographic separation system and employed as a detector for sulfur and copper. Oxygen was used as the reaction gas and sulfur was measured at a mass-to-charge ratio of 48 as SO^+^, whereas copper was measured simultaneously at a mass-to-charge ratio of 65. All sample dilutions were carried out in pre-cleaned polyethylene vials in Class 100 clean benches. Measurements were performed in a Class 10 000 clean room.

The generation and export of HPLC-ICPMS chromatograms were carried out using *ChromLink* (v.2.1; PE-SCIEX) in combination with *TotalChrom* (v.6.2; PE-SCIEX). Chromatographic data were processed using *Chromeleon* v.6.7 (Dionex, USA). The calibration of S and Cu and further calculations of the stoichiometric ratios of the two elements in the investigated proteins were performed using Cu/Zn superoxide dismutase (Sigma, USA).

### 2.5. Kinetics   

The laccase activity was measured at 30°C by monitoring the oxidation of either 2 m*M* ABTS at 420 nm (∊_420_ = 36 000 *M* ^−1^ cm^−1^) or of 1 m*M* 2,6-dimethoxyphenol (2,6-DMP) at 469 nm (∊_469_ = 49 600 *M* ^−1^ cm^−1^) in 100 m*M* sodium citrate buffer pH 4.0 (air-saturated). One unit of enzymatic activity was defined as the amount of enzyme required to obtain 1 µmol product per minute. The protein concentration of the homogeneous laccase solution was determined from the absorption at 280 nm using the calculated molar absorption coefficient ∊_280_ = 125 290 *M* ^−1^ cm^−1^. The pH profiles were determined for both substrates over the pH range 2.5–8 in 100 m*M* citrate–phosphate buffer. Kinetic constants were determined for both substrates in 100 m*M* citrate–phosphate buffer at pH 3.0, 4.0, 5.0 and 6.0. All kinetic experiments were performed in triplicate. The resulting curves were fitted to the Michaelis–Menten equation by nonlinear least-square regression with *SigmaPlot* 11.0 (Systat Software, Chicago, Illinois, USA).

### 2.6. Electrochemical measurements of the redox potential of the T1 copper site   

The redox potentials of the T1 sites of the two laccases were determined by mediated spectroelectrochemical redox titration (Christenson *et al.*, 2006 ▶). The home-built spectroelectrochemical cell consisted of a 1 cm long gold capillary electrode with an inner diameter of 350 µm, serving both as the working electrode and as a cuvette with a total volume of 1 µl. The input and output optical fibres (FCB-UV 400/050-2 and FC-UV 200, respectively) were purchased from Ocean Optics (Dunedin, Florida, USA) and attached to the ends of the capillary. The system comprised a DH-2000 light source and an HR4000CG-UV-NIR spectrometer (Ocean Optics). The spectra were recorded with the Ocean Optics *SpectraSuite* software. The potential of the gold capillary electrode was controlled using a μAutolabIII/FRA2 potentiostat/galvanostat (Metrohm Autolab, Utrecht, The Netherlands). Two platinum wires served as counter electrodes and an Ag|AgCl|3 *M* KCl electrode (210 mV *versus* NHE), separated from the enzyme solution by two ceramic frits and a buffer salt bridge, excluding chloride from the enzyme solution, was used as the reference electrode. The working gold capillary electrode was cleaned for approximately 10 h in freshly prepared 3:1(*v*:*v*) 96% sulfuric acid:30% H_2_O_2_ (piranha) solution.

50 µ*M* each of the reduced form of four redox mediators, K_4_[Fe(CN)_6_], K_4_[W(CN)_8_], K_4_[Os(CN)_6_] and K_4_[Mo(CN)_8_], with redox potentials equal to 430, 520, 640 and 780 mV *versus* NHE (Tsujimura *et al.*, 2005 ▶), were used to enhance communication between the enzymes and the electrode. A 50 µl aliquot of a laccase solution in 0.1 *M* phosphate buffer pH 5.5, also containing a mixture of redox mediators, was aspirated through the capillary to replace the buffer in the cell. The entire cell and all solutions were deoxygenated by flushing with argon (AGA GasAB, Sundbyberg, Sweden) before experiments. The redox potentials of the enzymes were determined by sequentially applying a series of potentials to the gold capillary electrode. Each potential was maintained until the Nernst equilibrium was reached (for approximately 5 min) between the oxidized and the reduced forms of the mediators, the enzyme and the poised electrode. The redox mediators were converted stepwise from one redox state to another by changing the applied potential, while the concentrations of the oxidized and reduced forms of the enzymes were determined from the spectra. Basic titration parameters, such as the midpoint potential at pH 5.5 (*E*_m5.5_), *b* (the slope of the titration curve), *n* (the number of electrons) and *R* (the correlation coefficient) were determined from plots of the applied potentials (*E*_appl_) *versus* log([Ox]/[Red]). All reported potentials are referred to NHE and all redox titrations were performed in 0.1 *M* phosphate buffer pH 5.5.

### 2.7. Deglycosylation   

Both laccase variants were deglycosylated using 1000 U mg^−1^ endoglycosidase Hf (New England BioLabs, USA) and 0.1 U mg^−1^ α-mannosidase (Sigma, USA) in 50 m*M* sodium citrate buffer pH 5.5 containing 10 m*M* ZnCl_2_ for 3 h at room temperature. The deglycosylation mixture was loaded onto a Superdex 75 gel-filtration column (GE Healthcare, USA) equilibrated with 50 m*M* sodium citrate buffer pH 5.5 to remove the deglycosylating enzymes. Pure fractions were concentrated and stored at 4°C. The deglycosylated forms of *Ba*L and L499M *Ba*L were only used for crystallization experiments.

### 2.8. Crystallization   

Crystallization conditions were screened by the sitting-drop vapour-diffusion method on a Rigaku Crystal Trak + Phoenix + Gallery 700 system (Rigaku, Japan) using the commercial high-throughput versions of the Index, Crystal Screen and PEG/Ion crystallization screens (Hampton Research, USA). The crystallization experiments were performed using solutions of *Ba*L and L499M *Ba*L at a concentration of 20 mg ml^−1^ in 25 m*M* sodium acetate buffer pH 5.0. Small crystals of deglycosylated *Ba*L were grown in conditions A4, A10, C8, D11 and E5 of Crystal Screen. Small crystals of deglycosylated L499M *Ba*L were obtained in condition F10 of Index and conditions A9, B8 and F3 of Crystal Screen.

The crystallization conditions were optimized using the hanging-drop vapour-diffusion technique at 298 K. The drops were composed of equal volumes (1 µl) of protein solution and reservoir solution. Crystals suitable for X-ray diffraction were obtained from reservoir solutions consisting of 1.8 *M* ammonium sulfate solution in water for deglycosylated *Ba*L and of 1.0 *M* lithium sulfate, 0.5 *M* ammonium sulfate, 0.1 *M* sodium citrate pH 5.5 for deglycosylated L499M *Ba*L. In both cases the crystals appeared within 3 d and reached maximum dimensions of 0.2 × 0.1 × 0.05 mm in one month. Crystallization of the glycosylated form of the wild-type enzyme was unsuccessful.

### 2.9. X-ray data collection and structure solution   

The X-ray data sets were collected on the K4.4e beamline at the Belok station at the Kurchatov synchrotron-radiation source at a wavelength of 0.98 Å using a Rayonix SX165 detector at 100 K under a nitrogen flow. For X-ray data collection under cryogenic conditions, the crystals were transferred to a harvesting solution composed of the reservoir solution with the addition of 20%(*v*/*v*) glycerol and were then flash-cooled. Both X-ray data sets were collected to 1.7 Å resolution and processed using the *XDS* program package (Kabsch, 2010 ▶). The data-collection statistics are summarized in Table 1 ▶. The crystals of *Ba*L and L499M *Ba*L belonged to space group *C*2 and had similar unit-cell parameters.

The structure of *Ba*L was solved by the molecular-replacement method using the fully automatic molecular-replacement pipeline *BALBES* (Long *et al.*, 2008 ▶). All other crystallographic calculations were carried out using the *CCP*4 program suite (Winn, 2011 ▶). The structures were refined with *REFMAC*5 (Murshudov *et al.*, 2011 ▶). The temperature factors were refined anisotropically for the Cu atoms and isotropically for all other non-H atoms. In the final steps of refinement, the occupancies of the copper ions were corrected manually assuming that the temperature factors of the copper ions should be approximately equal to the temperature factors of their ligands. Visual inspection and manual rebuilding of the models were carried out with the use of the *Coot* interactive graphics program (Emsley *et al.*, 2010 ▶). Water molecules were manually added to the structures based on the analysis of difference electron-density maps. The quality of the structures was evaluated with *SFCHECK* (Vaguine *et al.*, 1999 ▶) and *PROCHECK* (Laskowski *et al.*, 1993 ▶). Cruickshank’s DPI for coordinate error was calculated with *REFMAC*5. The molecular packing in the crystals was analyzed with *PISA* (Krissinel & Henrick, 2007 ▶). The figures were drawn with *CCP*4*mg* (McNicholas *et al.*, 2011 ▶). The structures have been deposited in the Protein Data Bank (PDB entries 3sqr and 3v9e for *Ba*L and L499M *Ba*L, respectively).

## 3. Results and discussion   

### 3.1. General characterization of laccase samples   

Samples of *Ba*L and L499M *Ba*L were purified to homogeneity (Supplementary Table S1 and Fig. S1). According to the SDS–PAGE data, the recombinantly produced *Ba*L and L499M *Ba*L have the same molecular mass of about 110 kDa. The amount of glycosyl residues (percentage of total mass) is identical for both enzymes (45%). In solution *Ba*L and L499M *Ba*L exist as dimers with a molecular mass of about 200 kDa (Supplementary Fig. S2).

No difference between the *Ba*L and L499M *Ba*L enzymes were observed in their CD spectra (Supplementary Fig. S3).

### 3.2. Redox potential, kinetic data and copper content   

The redox potential of the T1 site of *Ba*L was determined by spectroelectrochemistry to be 720 mV. The redox potential of the mutant is 140 mV lower (Supplementary Fig. S4).

**Figure 1:**
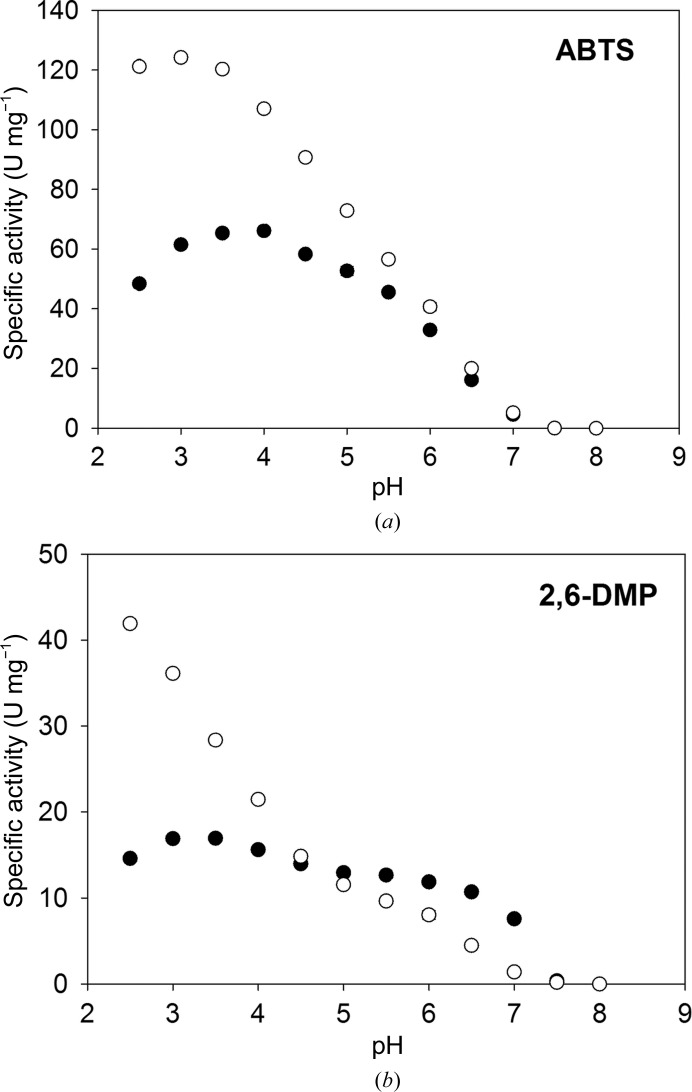
pH profiles of *Ba*L (empty circles) and L499M *Ba*L (filled circles) with the substrates (*a*) ABTS and (*b*) 2,6-DMP.

The pH dependencies of the specific activities of *Ba*L and L499M *Ba*L were determined using the substrates ABTS and 2,6-DMP (Fig. 1 ▶). The specific activity of L499M *Ba*L towards ABTS was lower than that of *Ba*L over the whole pH range and was lower for 2,6-DMP at pH < 5, which is consistent with the lower potential of the T1 site of L499M *Ba*L. However, in the pH range 5–7 L499M *Ba*L showed a higher activity than *Ba*L. The pH optima of the activities are acidic for both forms of the enzyme. However, the pH optimum for the mutant form is shifted to less acidic pH values: from pH 3 to pH 4 for ABTS and from pH 2.5 (or lower) to pH 3.5 for 2,6-DMP.

The catalytic constants were determined at pH 3.0, 4.0, 5.0 and 6.0 for ABTS and 2,6-DMP (Table 2 ▶). For ABTS the *V*_max_ values decrease and the apparent *K*_m_ values increase with increasing pH for both forms of the enzyme. This causes a 100-fold decrease in the efficiency of catalysis (*V*_max_/*K*_m_). In the case of the phenolic substrate 2,6-DMP, the *K*_m_ and *V*_max_ values decrease on increasing the pH from 3.0 to 6.0 for *Ba*L. For the mutant form the *V*_max_ remains relatively constant, causing the observed higher specific activity of L499M *Ba*L in the pH profile at elevated pH.

Freshly prepared homogeneous *Ba*L and L499M *Ba*L contained 2.6 and 2.5 copper ions per molecule, respectively, as determined from the Cu/S stoichiometry. During long-term storage of *Ba*L the copper content decreased to 2.3 copper ions per molecule, while the activity decreased from 100 to 9% (Supplementary Fig. S5).

### 3.3. Overall three-dimensional structure of laccase   

**Figure 2:**
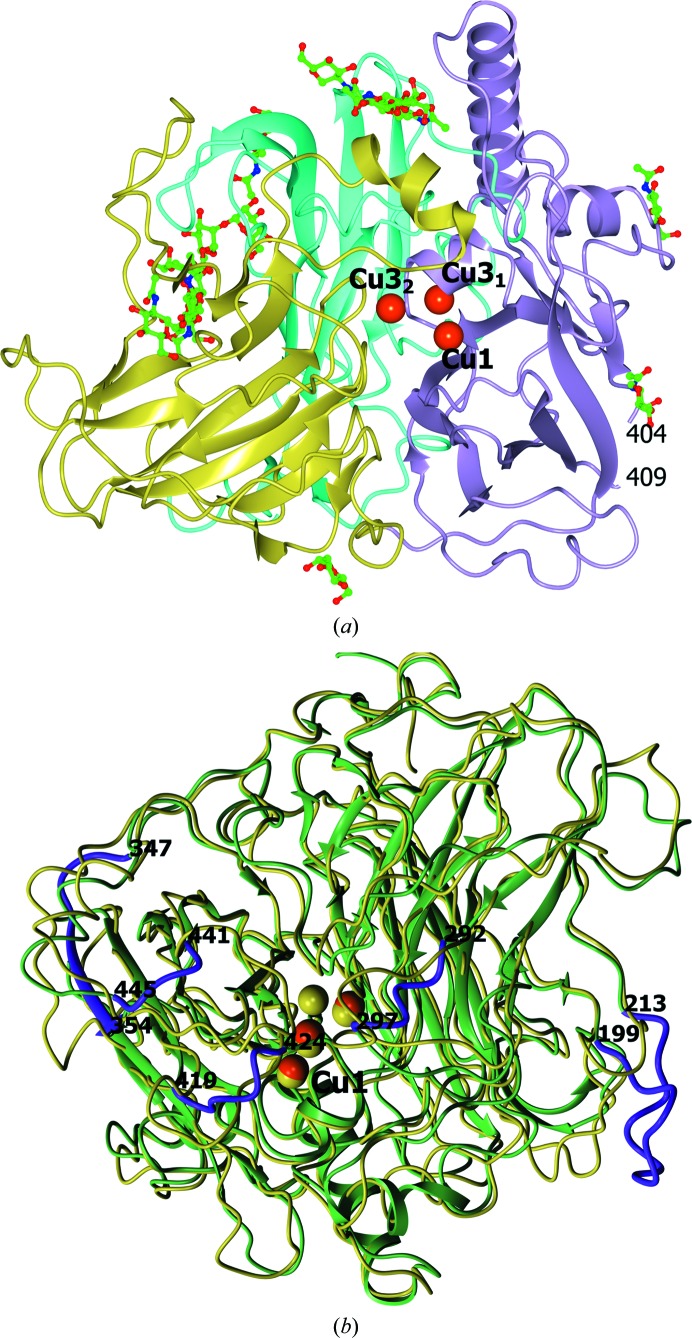
(*a*) Ribbon representation of the *Ba*L structure. Domains 1, 2 and 3 are shown in cyan, gold and violet, respectively. The copper ions are displayed as orange spheres. The carbohydrate residues are shown as ball-and-stick models coloured according to atom type. (*b*) The superimposed structures of *Ba*L (green) and *Ma*L (gold) using 402 pairs of equivalent C^α^ atoms. The copper ions are displayed as orange and gold spheres for *Ba*L and *Ma*L, respectively. In the *Ba*L structure, the loops which are most different from those in *Ma*L are shown in magenta.

The structures of *Ba*L and L499M *Ba*L were solved at 1.67 and 1.7 Å resolution, respectively. The structure of the monomer of *Ba*L is shown in Fig. 2 ▶(*a*). The enzyme consists of 543 amino-acid residues. Residues 405–408 were not located in the electron-density maps for both structures. The overall structure of L499M *Ba*L is similar to that of *Ba*L and can be superimposed with a C^α^ r.m.s.d. of 0.10 Å. In the Ramachandran plot, all residues lie in the allowed regions.

The overall fold of *Ba*L comprises three cupredoxin-like domains: 1 (1–151), 2 (152–343) and 3 (344–543). The active site is located between the first and third domains. The folding topology of the three-domain multicopper oxidases was analyzed for the first time by Messerschmidt *et al.* (1989 ▶). The characteristic structural feature of all laccases is a β-sandwich fold. The main structural difference between ascomycete laccases and laccases from other sources is that the N- and C-terminal regions of ascomycetous laccases include a larger number of amino-acid residues (Hakulinen *et al.*, 2002 ▶). The structure of *Ba*L could be superimposed with the structure of *Ma*L with an r.m.s.d. of 0.8 Å between 402 pairs of equivalent C^α^ atoms. The superimposed structures are shown in Fig. 2 ▶(*b*). The largest differences are observed in the arrangement of the loops (residues 199–213, 292–297, 347–354, 419–424 and 441–445). Some of them (292–297, 419–424 and 441–445) are in the substrate-binding pocket as described for *Ma*L (Kallio *et al.*, 2009 ▶.

The amino-acid sequence of *Ba*L contains nine cysteine residues, eight of which form disulfide bonds: 2–11, 297–331, 108–524 and 201–209. The first two bonds are structurally conserved for ascomycete laccases. The third bond is structurally conserved in all fungal laccases and stabilizes the arrangement of the C-terminal α-helix, which is absent in bacterial laccases. The ninth cysteine residue Cys489 is located in the active site.

### 3.4. Glycosylation in the structures of *Ba*L and L499M *Ba*L   

The laccase from the fungus *B. aclada* is highly glycosylated. Attempts to grow crystals of glycosylated forms of the enzyme failed. Prior to crystallization, *Ba*L and L499M *Ba*L were deglycosylated using α-mannosidase, which cleaves α(1→2,3,6) glycosidic bonds between mannose (MAN) residues, and endoglycosidase Hf, which catalyzes the cleavage of the β(1→6) glycosidic bond between *N*-β-d-acetyl­glucosamine (NAG) residues. Deglycosylation resulted in a decrease in the masses of *Ba*L and *Ba*L L499M from 110 to 70 kDa (Supplementary Fig. S1). Deglycosylated forms of *Ba*L and L499M *Ba*L retained approximately 70% of the initial activity of freshly prepared *Ba*L and L499M BaL, respectively (data not shown).

Nine potential *N*-glycosylation sites were detected following the canonical N-*X*-S/T sequence motif in the amino-acid sequence of *Ba*L. Seven of them were actually glycosylated: 39, 55, 82, 194, 305, 370 and 389. Nine NAG residues and six MAN residues were located in the electron-density map for *Ba*L, and nine NAG residues and three MAN residues were located in that for L499M *Ba*L. One NAG residue was attached to each of the five Asn residues (39, 55, 305, 370 and 389). A difference in the residual glycosylation between *Ba*L and L499M *Ba*L was found in the carbohydrate chain attached to Asn194 (see below).

**Figure 3:**
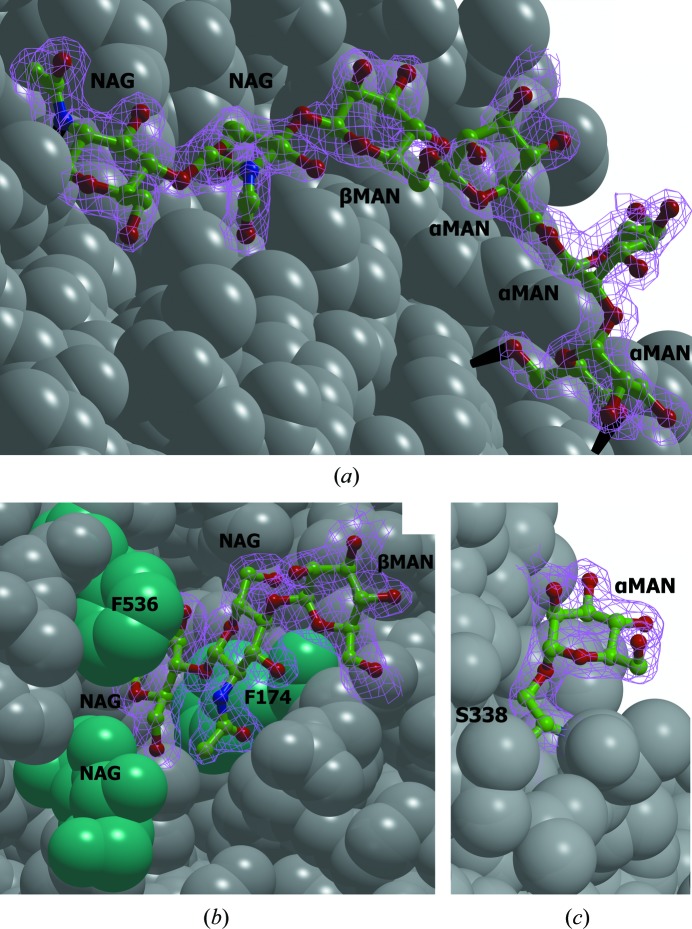
Glycosylation of *Ba*L. (*a*) The carbohydrate chain attached to Asn194; (*b*) the carbohydrate chain attached to Asn82; (*c*) the α-mannose residue attached to Ser338. The carbohydrate chains are shown as ball-and-stick models. The protein surface is represented by grey balls (the Phe174 and Phe536 residues and the NAG residue attached to Asn39 are in cyan). The 2*F*_o_ − *F*_c_ electron-density maps (at the 1σ level) are shown in magenta. Hydrogen bonds are shown as black lines.

In the structure of *Ba*L the carbohydrate chain at Asn194 contains six carbohydrate residues: MAN(α1→2)MAN(α1→6)MAN(α1→6)MAN(β1→4)NAG(β1→4)NAG(β1→N^δ^)Asn194 (Fig. 3 ▶ *a*). The terminal α-MAN residue (MAN557) in this chain forms two hydrogen bonds (MAN O6⋯Tyr33 OH and MAN O4⋯Glu32 N). This results in the stabilization of the orientation of this residue and can hinder its cleavage by α-mannosidase. The resistance of the NAG(β1→4)NAG bond in this chain to deglycosylation is attributed to the following factors: the location of this chain in the cavity on the protein surface and the stabilization of its conformation through hydrogen bonding between the terminal α-MAN residue and the first NAG residue (NAG O3⋯Val160 O). Owing to the stabilization of the conformation of the carbohydrate, the *B* factors of the terminal carbohydrate residues are much lower than those of the central residues. In the L499M *Ba*L structure, the corresponding carbohydrate chain is shorter and only three residues [MAN(β1→4)NAG(β1→4)NAG(β1→N^δ^)Asn194] were located in the electron-density map.

The carbohydrate chain at Asn82 contains three carbo­hydrate residues: MAN(β1→4)NAG(β1→4)NAG(β1→N^δ^)Asn82 (Fig. 3 ▶ *b*). This chain is located in the cavity on the protein surface, and access to the glycosidic bond between the NAG residues is blocked by the side chains of Phe174 and Phe536 and the NAG residue attached to Asn39. The resistance of the bond between the NAG residues in the carbohydrate chain at Asn82 to deglycosylation by endoglycosidase Hf is owing to steric hindrance. The presence of a β-MAN residue in this chain is attributed to the fact that the enzyme specific for the MAN(β1→4)NAG glycosidic bond was not used in deglycosylation.

The Ser338 residue in the *Ba*L and L499M *Ba*L structures is *O*-glycosylated [MAN(α1→O^γ^)Ser; Fig. 3 ▶ *c*], which is not typical of fungal laccases. This is apparently owing to the expression of the protein in the yeast *P. pastoris* (Cereghino & Cregg, 2000 ▶).

The hydrogen bonds of some carbohydrate residues stabilize the crystal structure through intermolecular hydrogen bonding. Thus, the NAG residue attached to Asn55 forms four hydrogen bonds to the Glu38, Ser40 and Thr41 residues of the adjacent molecule related to the original molecule by the symmetry operation (−*x*, *y*, −*z* − 1). The NAG residue attached to Asn389 forms two hydrogen bonds to Glu521 of the adjacent molecule related to the original molecule by the symmetry operation (−*x*−1, *y*, *z* − 1).

### 3.5. Dimeric structure   

Gel filtration showed that the deglycosylated forms of *Ba*L and L499M *Ba*L, like the glycosylated forms, exist as dimers in 25 m*M* sodium acetate pH 5.0 (Supplementary Fig. S2).

**Figure 4:**
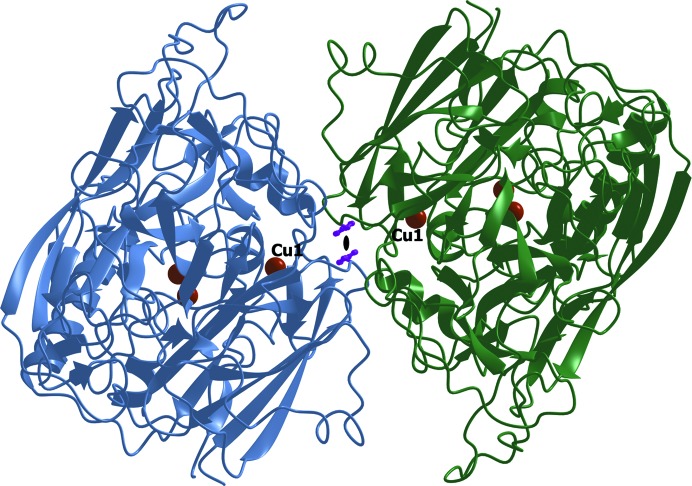
The *Ba*L dimer in the crystal. The side chains of the Phe422 residues in both molecules are shown as purple ball-and-stick models and the copper ions are displayed as orange spheres. The crystallographic twofold rotation axis is perpendicular to the plane of the figure and is shown as a black lens-shaped symbol.

An analysis of the molecular packing of *Ba*L in the unit cell using *PISA* revealed two major intermolecular contacts in the crystal. The first contact is characterized by the following parameters: an interface area *S* = 1007 Å^2^ and a free-energy gain upon formation of the interface Δ*G* = −13.1 kcal mol^−1^. The corresponding values for the second contact are *S* = 606 Å^2^ and Δ*G* = −10.1 kcal mol^−1^. The first contact involves the substrate-binding site. Although *PISA* predicts that the dimer stabilized *via* the first contact is unstable in aqueous solution, this dimer is of interest because similar dimers were found in the structures of the ascomycetous *Ma*L (Hakulinen *et al.*, 2002 ▶) and *Ta*L (Kallio *et al.*, 2011 ▶). The dimeric contact in *Ba*L has a slightly larger surface and a higher energy of formation compared with the structures of *Ma*L (*S* = 797 Å^2^, Δ*G* = −12 kcal mol^−1^) and *Ta*L (*S* = 677 Å^2^, Δ*G* = −11 kcal mol^−1^). The subunits in the dimers of ascomycete laccases are related by a twofold rotation axis (in the *Ba*L structures this is a crystallographic axis, while in the *Ma*L and *Ta*L structures it is a noncrystallographic axis). The dimer of *Ba*L is shown in Fig. 4 ▶. Residues 182–187, 295–304, 358–367, 418–425, 442–444 and 469–470 are at the dimer interface. The dimer is formed through hydrophobic interactions, stacking interactions between the Phe422 residues and two hydrogen bonds between Asn470 N^δ^ and Thr296 O in the two subunits of the dimer. The Phe422 residue, which is involved in the substrate-binding site and is located at the dimer interface, is at a distance of 3.7 Å from Phe422 in the adjacent molecule. The planes of the benzene rings of Phe422 are coplanar, and the distance between the normals to the centroids of the rings is 1.3 Å, which corresponds to a strong π–π interaction between Phe422 (Hunter *et al.*, 1991 ▶; Hunter & Sanders, 1990 ▶). The dimers of *Ma*L and *Ta*L are almost identical. They are formed through hydrophobic interactions and six hydrogen bonds. The differences in the packing of the *Ba*L and *Ma*L or *Ta*L dimers are determined mainly by the differences in the structures of the loops 292–297, 419–424 and 441–445 in the region of the substrate-binding site (near the T1 copper site; Fig. 2 ▶ *b*). As a result of these differences, the distance between Cu1 in the *Ba*L dimer is 20 Å, while the corresponding distance in *Ma*L and *Ta*L is 27 Å.

### 3.6. Active site   

The active site of *Ba*L contains three copper ions in two sites (T1 and T2/T3).

**Figure 5:**
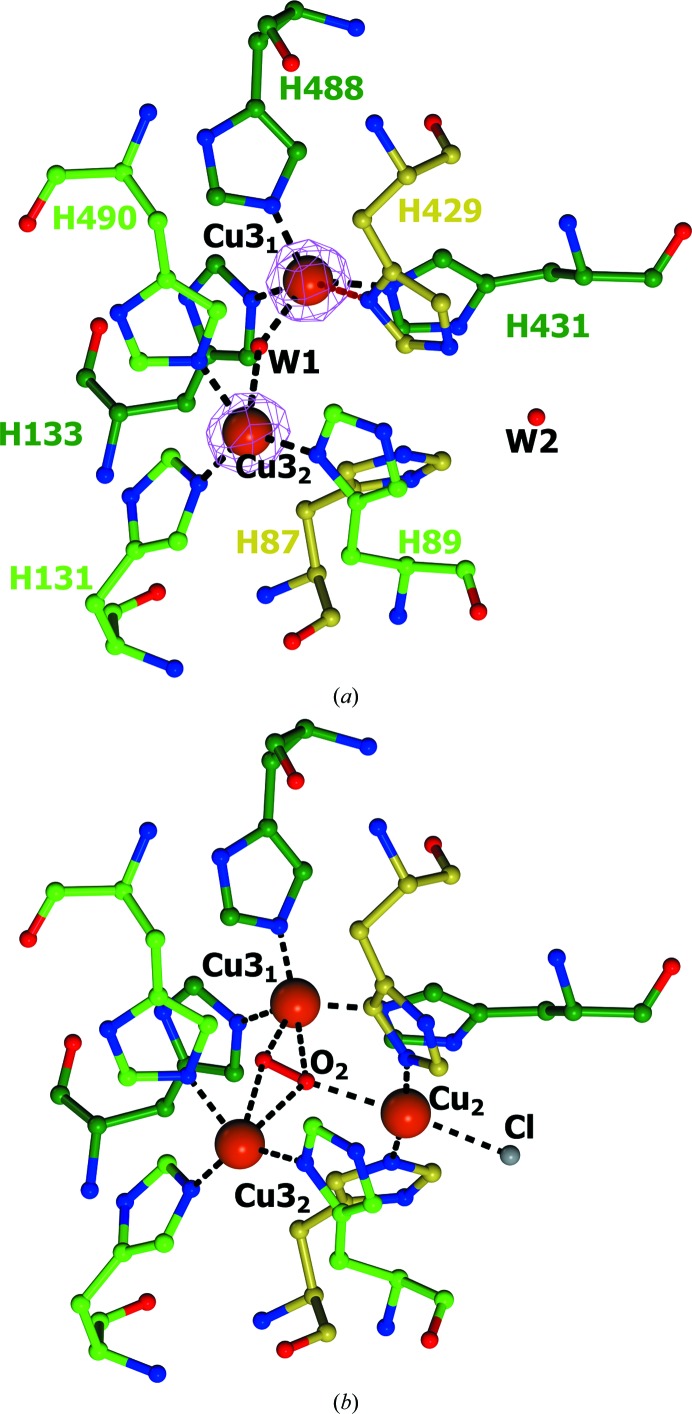
Structures of the T2/T3 copper sites in (*a*) *Ba*L and (*b*) *Ma*L. The amino-acid residues are shown as ball-and-stick models and are coloured according to atom type. The C atoms of the His residues coordinated to the Cu3_1_, Cu3_2_ and Cu2 ions are shown in dark green, light green and gold, respectively. The oxygen ligands and the chloride ion are displayed as ball-and-stick models in red and grey, respectively. The copper ions are represented as orange spheres. The 2*F*_o_ − *F*_c_ electron-density map (at the 1.0σ level) is shown in magenta. Coordination bonds are indicated by black dashed lines. The weak coordination bond between Cu3_1_ and His429 N^δ^ is shown as a violet dashed line. The structure of *Ma*L from PDB entry 2q9o was used (Hakulinen *et al.*, 2002 ▶).

The T2/T3 copper site includes two type 3 copper ions (Cu3_1_ and Cu3_2_) and eight histidine residues (Fig. 5 ▶ *a*). The coordination of both type 3 copper ions can be described as a distorted tetrahedron. The structure of the T2/T3 copper site in *Ba*L is identical to that in L499M *Ba*L. The main distinguishing feature of the T2/T3 copper site in the structures of *Ba*L is that they do not contain the type 2 copper ion, *i.e.* the enzymes are T2 copper-depleted.

**Figure 6:**
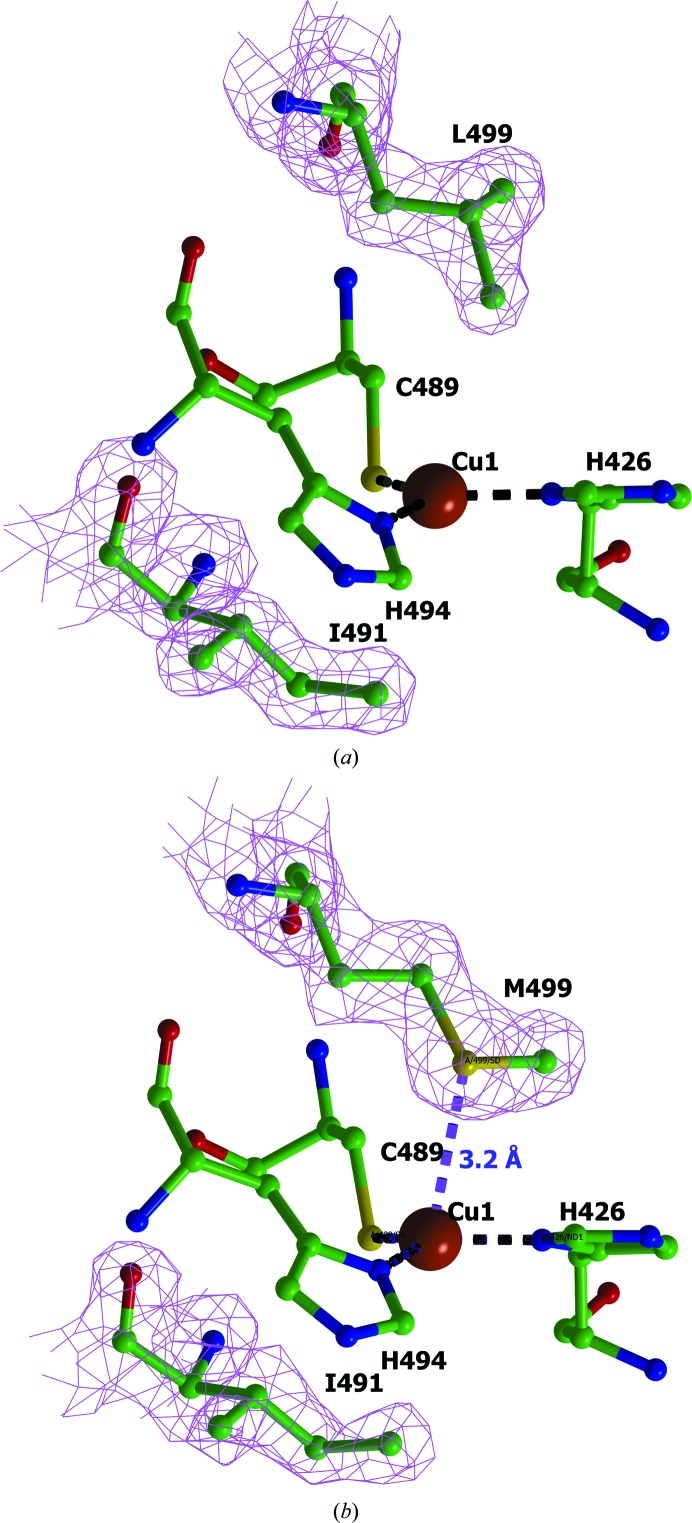
Structure of the T1 copper site in (*a*) *Ba*L and (*b*) L499M *Ba*L. The 2*F*_o_ − *F*_c_ electron-density maps for the axial residues are shown at the 1σ level. The amino-acid residues are displayed as ball-and-stick models and are coloured according to atom type. The copper ions are represented by orange spheres. The coordination bonds at the Cu1 ion are indicated by black dashed lines. The weak coordination bond between Met499 S^δ^ and Cu1 is shown in purple with the bond length on the right.

The T1 copper site is formed by the type 1 copper ion (Cu1) coordinated by the side chains of two histidine residues (His426 and His494) and the S atom of Cys489 to form a trigonal planar geometry (Fig. 6 ▶ *a*). The side chains of the hydrophobic amino-acid residues are located on both sides of this plane. The Ile491 residue is conserved in both structures. The Leu499 residue in *Ba*L is replaced by a methionine in L499M *Ba*L (Fig. 6 ▶ *b*). The T1 copper site is connected to the T2/T3 copper site through the conserved HCH motif, with the histidine residues being coordinated to Cu3_1_ and Cu3_2_, and cysteine being coordinated to Cu1. By assuming that the *B* factors of the ligands and the central metal atoms should be similar, the Cu1 site was refined with full occupancy and the Cu3_1_ and Cu3_2_ sites were refined with occupancies of 0.8.

In both structures we do not observe the Cu2 ion and the sum of copper occupancies over all copper sites gives 2.6 copper ions per molecule. The copper content in the structures is in good agreement with the copper content determined in solutions of freshly prepared samples. It should be noted that a small decrease in the copper content observed for enzyme samples after storage is accompanied by a dramatic decrease in activity. This observation, combined with the fact that the inactive T2-depleted form of the enzyme is present in the crystal, suggests that the loss of the activity during storage and crystallization is primarily owing to a decrease in the occupancy of the T2 site.

### 3.7. Structure of the T2/T3 copper site   

The structure of the T2/T3 copper site in *Ba*L is shown in Fig. 5 ▶(*a*). The distances between the copper ions and the atoms coordinated to these ions are given in Table 3 ▶. The Cu3_1_ ion is coordinated by the N^∊^ atoms of His133, His431 and His488, and the water molecule shared by Cu3_1_ and Cu3_2_ (W1; Fig. 5 ▶ *a*). The Cu3_2_ ion is coordinated by the N^δ^ atom of His89, the N^∊^ atoms of His131 and His490, and the water molecule W1. The distance between the copper ions of the T3 site is 4.6 Å.

There are the following main differences in the structures of the active sites of *Ba*L and *Ma*L (Fig. 5 ▶ *b*). Firstly, no electron density for Cu2 was found between His87 and His429, which is evidence that the type 2 copper ion is absent in *Ba*L (both forms of the enzyme gradually lose activity during storage; Supplementary Fig. S5).

Secondly, the imidazole ring of His429 is flipped compared with the equivalent His434 (which is coordinated to Cu2) in the *Ma*L structure. The N^δ^ atom of His429 is involved in an additional weak coordination bond to Cu3_1_ (Table 3 ▶), and the N^∊^ atom forms a hydrogen bond to the water molecule W2.

The T2 copper ion is completely absent in the previously described structures of the laccases from *C. cinereus* (Ducros *et al.*, 1998 ▶, 2001 ▶) and *Thermus thermophilus* (De la Mora *et al.*, 2012 ▶). In these structures, the histidine residue of the T2 site related to His429 in the *Ba*L structure adopts two alternative orientations. In one of these orientations the histidine can coordinate Cu2, whereas in the other orientation it forms a weak coordination bond to Cu3_1_ through the N^∊^ atom. Structures in which the T2 copper site is partially occupied (with an occupancy of 0.2) are also known (PDB entries 3v9c and 3pxl; K. M. Polyakov, T. V. Fedorova, S. A. Kurzeev, A. N. Popov, V. S. Lamzin & O. V. Koroleva, unpublished work). In these structures, the histidine residue equivalent to His429 in the *Ba*L structure also has two alternate conformations and either coordinates Cu2 or forms a weak coordination bond with Cu3_1_ through the N^δ^ atom by analogy with the *Ba*L structure. Therefore, the absence of Cu2 in the *Ba*L structures is shown to be ultimately associated with the rotation of His429 towards the Cu3_1_ copper ion.

### 3.8. Structure of the T1 copper site and its redox potential   

The structure of the T1 copper site in *Ba*L and L499M *Ba*L is shown in Fig. 6 ▶. The interatomic distances in the T1 site are given in Table 3 ▶. In *Ba*L the axial positions at the Cu1 ion are occupied by the isoleucine residue that is conserved in all wild-type three-domain laccases and by the leucine residue.

In *Ba*L, as in most of the known laccases, the Cu1 ion lies in the plane formed by the coordinated atoms of histidine and cysteine residues. In the T1 site of L499M *Ba*L, Cu1 is displaced from this plane towards the S^δ^ atom of Met499 by 0.17 Å. This value is within the experimental error (Table 1 ▶). A similar small deviation of Cu1 from the plane is observed in several laccase structures with an axial methionine residue, such as *B. subtilis* (Enguita *et al.*, 2003 ▶), *Amycolatopsis* sp. ATCC 39116 (PDB entry 3t9w; Majumdar *et al.*, 2014 ▶), *Campylobacter jejuni* (Silva *et al.*, 2012 ▶) and a metagenome-derived laccase (Komori *et al.*, 2009 ▶), as well as ascorbate oxidase (Messerschmidt *et al.*, 1989 ▶). In all of these structures there is a short distance between Cu1 and the S^δ^ atom of the axial methionine residue (2.8–3.3 Å), which attests to the presence of a weak coordination bond. In the L499M *Ba*L structure the distance between Cu1 and the S^δ^ atom of Met499 is 3.2 Å, which also can be ascribed to a weak coordination bond. However, in the laccase from *T. thermophilus*, which contains an axial methionine, no deviation of the copper ion from the plane is observed and the distance between Cu1 and the S^δ^ atom of the axial methionine is about 3.6 Å.

Table 4 ▶ presents the redox potentials of a number of laccases and ascorbate oxidase with known three-dimensional structures (we chose representatives from each group of structurally similar enzymes). In addition, Table 4 ▶ summarizes the data on the effect of mutations in the environment of the T1 copper site on the redox potential of laccases. It should be noted that the redox potentials given in Table 4 ▶ were measured by different authors under different conditions. Hence, it is difficult to compare the absolute values of the redox potentials of different laccases. Moreover, two different values of the redox potential have been reported for the laccase from *B. subtilis* (Durão *et al.*, 2006 ▶, 2008 ▶).

Nevertheless, there is a correlation between the type of axial residue and the redox potential. Phenylalanine, leucine or methionine were found as the nonconserved axial residue. The effect of the type of nonconserved axial residue on the redox potential was studied for Met/Phe and Met/Leu mutants of the laccase from *B. subtilis* (Durão *et al.*, 2006 ▶) and Phe/Met and Phe/Leu mutants of the laccase from *T. villosa* (Xu *et al.*, 1999 ▶). The redox potentials of the mutant enzymes containing phenylalanine or leucine as the axial ligand are approximately equal to or higher than those of the methionine-containing mutants. The same situation is observed for all laccases. A comparison of the structures of *Ba*L and L499M *Ba*L shows that there are no changes in the structure of the enzyme except for the region near the axial mutation. Hence, the 140 mV decrease in the redox potential of L499M *Ba*L can be attributed exclusively to the mutation. The reverse substitution of the axial ligand (Met/Leu) in the laccase from *B. subtilis* led to a 90 mV increase in the redox potential with retention of the structure (Durão *et al.*, 2006 ▶).

The effect of the replacement of isoleucine in the axial position by alanine on the redox potential of the laccase from *B. subtilis* has been studied previously (Durão *et al.*, 2008 ▶). In the structure of the mutant form the position of the side chain of isoleucine is occupied by a water molecule, which forms an additional weak coordination bond to Cu1, resulting in a 100 mV decrease in the redox potential of the mutant form of the enzyme. It can be suggested that the main function of the axial residues is to create a hydrophobic environment of Cu1. Phenylalanine and leucine successfully fulfill this role, whereas methionine is less efficient because its S^δ^ atom can interact with Cu1 and owing to the higher conformational flexibility of the methionine side chain.

The nature of the axial ligands is not the only factor that influences the redox potentials of laccases. A substantial difference in the redox potentials of enzymes containing the same axial ligands can be attributed to the dependence of the redox potential on the more distant environment of the T1 site (the second coordination sphere). The effect of mutations in the second coordination sphere on the redox potential has been studied by site-directed mutagenesis (Durão *et al.*, 2008 ▶). The replacement of leucine, which shields the axial methionine residue from the solvent, in the laccase from *B. subtilis* led to a 60 mV decrease in the redox potential of the enzyme. The influence of the second coordination sphere on the redox potential can be estimated based on the data for *Ba*L and *Ma*L. Both of them have a similar amino-acid composition in the first coordination sphere, the same axial ligands in the T1 site and similar polypeptide chain folds. However, the difference between their redox potentials is 250 mV. Such changes in the redox potential of these enzymes can be associated with the differences in the structures of the loops 292–297, 419–424 and 441–445 (Fig. 2 ▶ *b*) located near the T1 site and the putative binding site of the phenolic substrate, as well as with the point substitutions resulting in an increase in the hydrophobicity of the environment of the T1 site in *Ba*L.

## Supplementary Material

PDB reference: *Botrytis aclada* laccase, 3v9e

PDB reference: L499M mutant, 3sqr

Supporting Information.. DOI: 10.1107/S1399004714020380/lv5066sup1.pdf

## Figures and Tables

**Table 1:** Data-collection and refinement statistics Values in parentheses are for the last resolution shell.

	*Ba*L	L499M *Ba*L
Data collection		
Space group	*C*2	*C*2
Unit-cell parameters (, )	*a* = 69.67, *b* = 113.50, *c* = 79.91, = 108.75	*a* = 69.71, *b* = 113.56, *c* = 79.98, = 108.74
Resolution ()	301.67 (1.771.67)	301.70 (1.801.70)
*I*/(*I*)	16.1 (2.1)	16.1 (2.8)
Completeness (%)	96.3 (90.6)	97.4 (95.2)
Total reflections	181887 (24180)	199830 (31000)
Unique reflections	65731 (9904)	62839 (9875)
Multiplicity	2.77 (2.44)	3.18 (3.14)
*R*_meas_ † (%)	4.7 (53.1)	6.5 (54.0)
Refinement		
*R*_cryst_ (%)	16.6	16.5
*R*_free_ (%)	19.1	20.0
Cruickshank’s DPI ()	0.10	0.11
R.m.s.d. from ideal values		
Bond lengths ()	0.021	0.017
Bond angles ()	1.91	1.97
No. of atoms		
Protein	4206	4187
Cu	3	3
Solvent	445	305
Other	197	164
Ramachandran plot statistics (%)		
Most favoured	86.7	86.7
Additionally allowed	12.7	13.1
Generously allowed	0.6	0.2
Disallowed	0	0
Average *B* factors (^2^)		
Protein	28.3	23.4
Solvent	34.7	28.1

† *R*_meas_ = 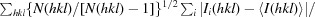 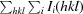, where *I_i_*(*hkl*) is the intensity of the *i*th observation with indices *hkl*, *I*(*hkl*) is the mean intensity of this reflection and *N*(*hkl*) is the multiplicity of the reflection with indices *hkl* (Diederichs Karplus, 1997 ▶).

**Table 2:** Kinetic constants for *Ba*L and L499M *Ba*L with ABTS and 2,6-DMP at acidic pH values

		*Ba*L		L499M *Ba*L	
Substrate	pH	*K*_m_ (*M*)	*V*_max_ (Umg^1^)	*K*_m_ (*M*)	*V*_max_ (Umg^1^)
ABTS	3.0	5.3 0.7	160.4 4.2	8.8 0.5	82.5 1.2
	4.0	14.0 1.0	126.9 2.6	19.3 1.0	74.2 1.1
	5.0	45.1 1.1	81.5 0.6	84 4	61.6 1.0
	6.0	148 4	41.8 0.5	500 19	40.4 0.6
2,6-DMP	3.0	41.8 2.7	52.4 1.0	420 18	27.1 0.4
	4.0	8.3 0.7	37.4 0.7	80.8 2.7	23.6 0.2
	5.0	2.2 0.3	25.0 0.4	35.1 1.4	23.0 0.2
	6.0	1.8 0.2	17.8 0.2	34.7 1.1	22.8 0.2

**Table 3:** Interatomic distances in the active sites of *Ba*L, L499M *Ba*L and *Ma*L The temperature factors of the atoms (^2^) in the structure of *Ba*L are given in parentheses.

Atom *A*	Atom *B*	Distance *A* *B* ()		
		*Ba*L	L499M *Ba*L	*Ma*L†
Cu1 (25.4)	Cys489S (23.4)	2.2	2.2	2.2
	His426N (22.1)	2.0	2.0	2.0
	His494N (23.2)	2.0	2.0	2.0
	Ile491C (22.7)	3.5	3.7	3.6
	Leu499C (24.9)	3.6		3.8
	Met499‡S		3.2	
Cu3_1_ (27.3)	His133N (23.0)	2.1	2.2	2.1
	His431N (21.4)	2.0	2.0	2.0
	His488N (25.6)	2.0	2.0	2.1
	W1 (31.2)	2.1	2.0	2.2§
	His429N (29.2)	2.8	2.6	6.2
Cu3_2_ (27.5)	His89N(21.7)	2.0	1.9	2.0
	His131N (22.6)	2.0	2.0	2.0
	His490N (24.0)	2.0	2.3	2.0
	W1 (31.2)	2.6	2.9	2.6§
	Cu3_1_ (27.3)	4.6	4.6	4.7

† For the *Ma*L structure, the interatomic distances between the copper ions and the ligands related to those in the *Ba*L structure are given.

‡ The S atom of the axial methionine in L499M *Ba*L.

§ The distances to the O atom equivalent to the water molecule W1 in the *Ba*L structure are given.

**Table 4:** Redox potentials of the T1 copper site in laccases and ascorbate oxidase from different sources All enzymes are laccases except for that from *Curcubita pepo* var. *cylindrica*, which is an ascorbate oxidase.

Source organism/mutation	Axial residues	Redox potential (mV)	Reference
Ascomycetes			
*B. aclada*	Leu, Ile	720	This study
*B. aclada*, L499M mutant	Met, Ile	580	This study
*M. albomyces*	Leu, Ile	470	Hakulinen *et al.* (2002 ▶)
*T. arenaria*	Leu, Ile	510	Kallio *et al.* (2011 ▶)
Basidiomycetes			
*T. hirsuta*	Phe, Ile	780	Kojima *et al.* (1990 ▶)
*T. villosa*	Phe, Ile	790	Xu *et al.* (1999 ▶)
*T. villosa*, F463L mutant	Leu, Ile	740	Xu *et al.* (1999 ▶)
*T. villosa*, F463M mutant	Met, Ile	680	Xu *et al.* (1999 ▶)
Bacteria			
*B. subtilis*	Met, Ile	455	Duro *et al.* (2006 ▶)
		525	Duro *et al.* (2008 ▶)
*B. subtilis*, I494A mutant	Met, Ala	429	Duro *et al.* (2008 ▶)
*B. subtilis*, L386A mutant	Met, Ile	466	Duro *et al.* (2008 ▶)
*B. subtilis*, M502L mutant	Leu, Ile	548	Duro *et al.* (2006 ▶)
*B. subtilis*, M502F mutant	Phe, Ile	515	Duro *et al.* (2006 ▶)
*C. jejuni*	Met, Ile	420	Silva *et al.* (2012 ▶)
Plant			
*Curcubita pepo* var. *cylindrica* (zucchini)	Met, Ile	350	Kroneck *et al.* (1982 ▶)

## Footnotes

1 Supporting information has been deposited in the IUCr electronic archive (Reference: LV5066).
