# Supplementary material for: Effect of the L499M mutation of the ascomycetous Botrytis aclada laccase on redox potential and catalytic properties
Source: Acta Crystallogr D Biol Crystallogr. 2014 Oct 23;70(Pt 11):2913–23. doi: 10.1107/S1399004714020380 (PMC4220974; doi:10.1107/S1399004714020380)
Supplement: Supplementary file 1 [file d-70-02913-sup1.pdf]

Acta Crystallographica Section D

Volume 70 (2014)

Supporting information for article:

Effect of the L499M mutation of ascomycetous *Botrytis aclada* laccase on redox potential and catalytic properties

Evgeny Osipov, Konstantin Polyakov, Roman Kittl, Sergey Shleev, Pavel Dorovatovsky, Tamara Tikhonova, Stephan Hann, Roland Ludwig and Vladimir Popov

Table S1 Purification scheme of *Ba*L and L499M *Ba*L

		Volume (ml)	Total activity (U)	Total protein (mg)	Specific activity (U mg^{-1})	Yield (%)
BaL	Culture supernatant	3000	117600	2310	51	100
	Phenyl-sepharose	120	85900	695	124	73
	Phenyl-source	70	47100	338	139	40
	Sephacryl S 300 HR	1,5	1820	11,6	158	2
L499M *Ba*L	Culture supernatant	2900	292000	5890	50	100
	Phenyl-sepharose	185	128300	1540	83	44
	Phenyl-source	60	45300	477,8	95	16
	Sephacryl S 300 HR	1,5	1620	16,2	100	0,6

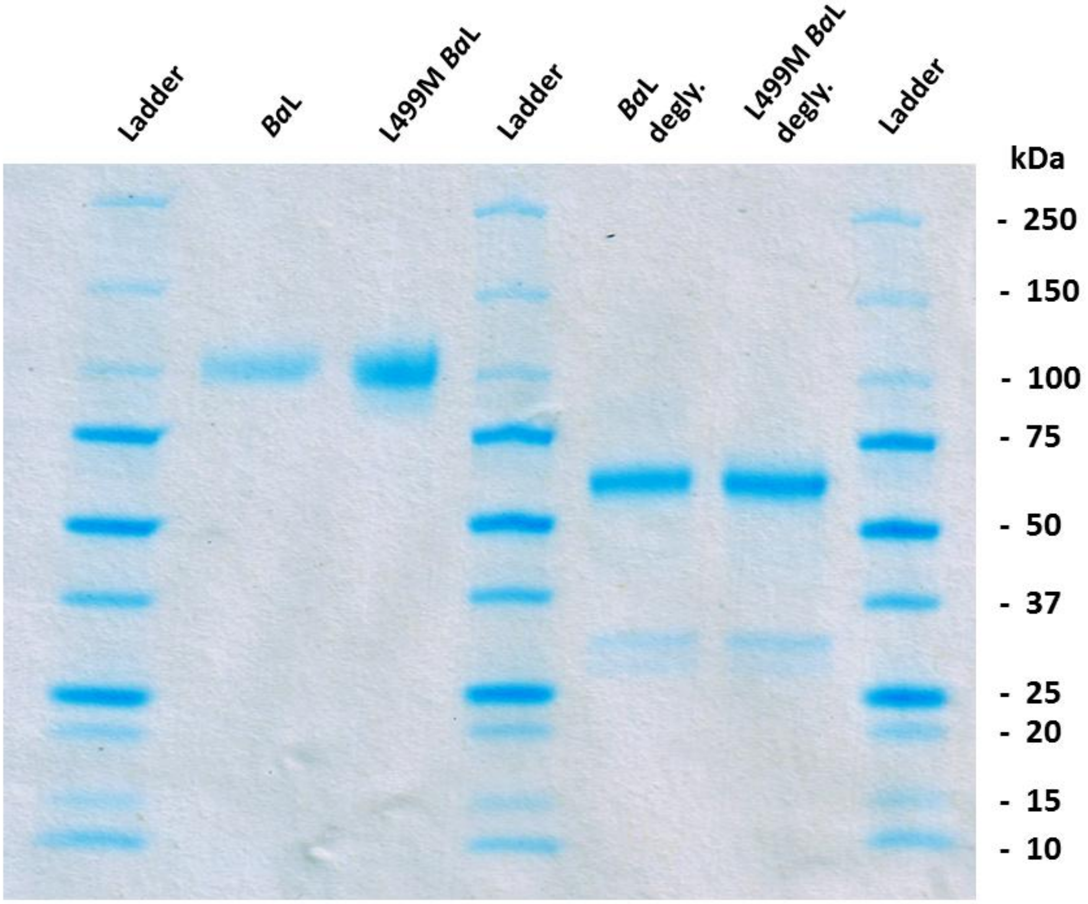

Figure S1 SDS-PAGE of BaL (2nd lane), L499M BaL (3rd lane), and the corresponding deglycosylated forms (5th and 6th lanes). The low molecular mass bands below the deglycosylated enzymes are from PNGase enzymes used for deglycosylation. Mini-PROTEAN TGX precast gels (Bio-Rad Laboratories) with a gradient from 4 – 15% were used. Protein bands were visualized by staining with Bio-Safe Coomassie, and unstained Precision Plus Protein Standard was used for mass determination. All procedures were done according to the manufacturer's recommendations (Bio-Rad Laboratories).

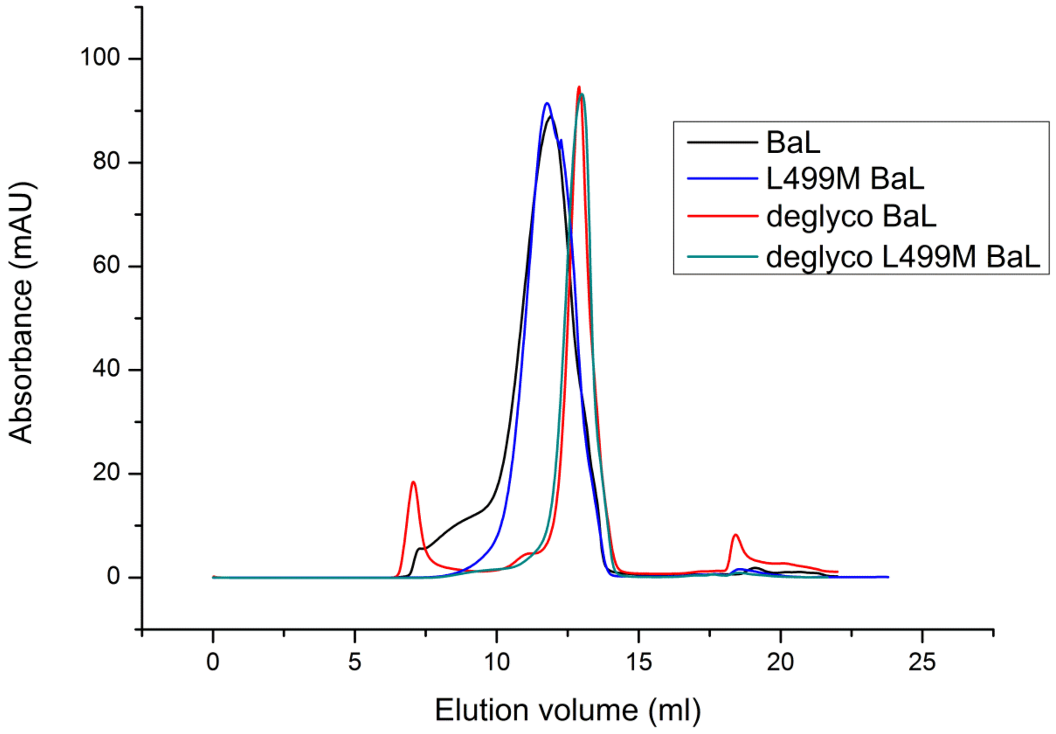

Figure S2 Gel filtration chromatograms of *Ba*L and L499M BaL. All curves refer to Superdex 200 10/300 GL equilibrated with 25 mM acetate buffer, pH 5.0. Both glycosylated forms elute as dimers with molecular mass ~ 180-200 kDa and both deglycosylated forms elute as dimers with molecular mass ~ 120-140 kDa.

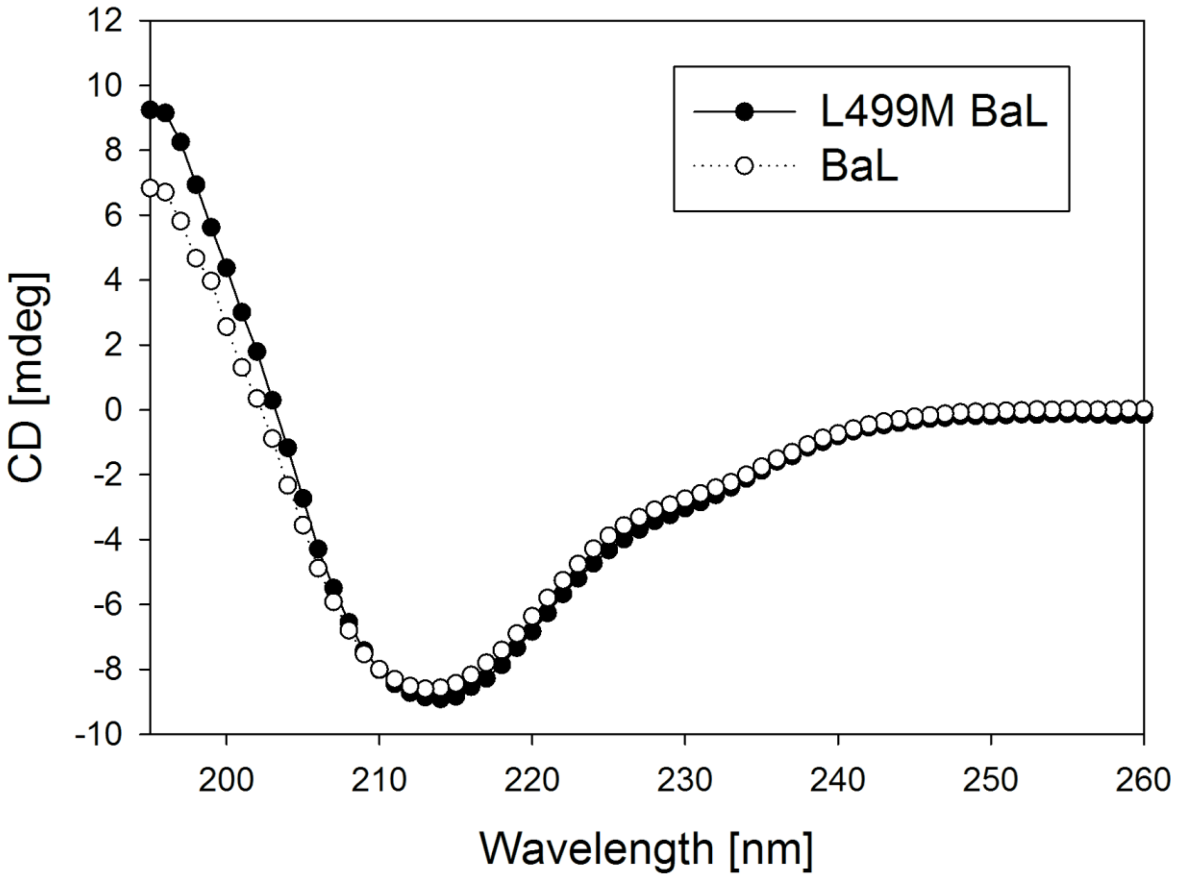

Figure S3 CD spectra recorded for *Ba*L (empty circles) and L499M BaL (filled circles). CD spectra were measured at room temperature using a Chiroscan CD Spectrophotometer (Applied Photophysics, Leatherhead, UK). Measurements were carried out using a 1 mm path-length cell and protein concentrations of 0.125 mg mL^{-1} with a scan rate of 6 nm min^{-1}. The sampling wavelength was set at 1 nm and the spectral bandwidth at 3 nm.

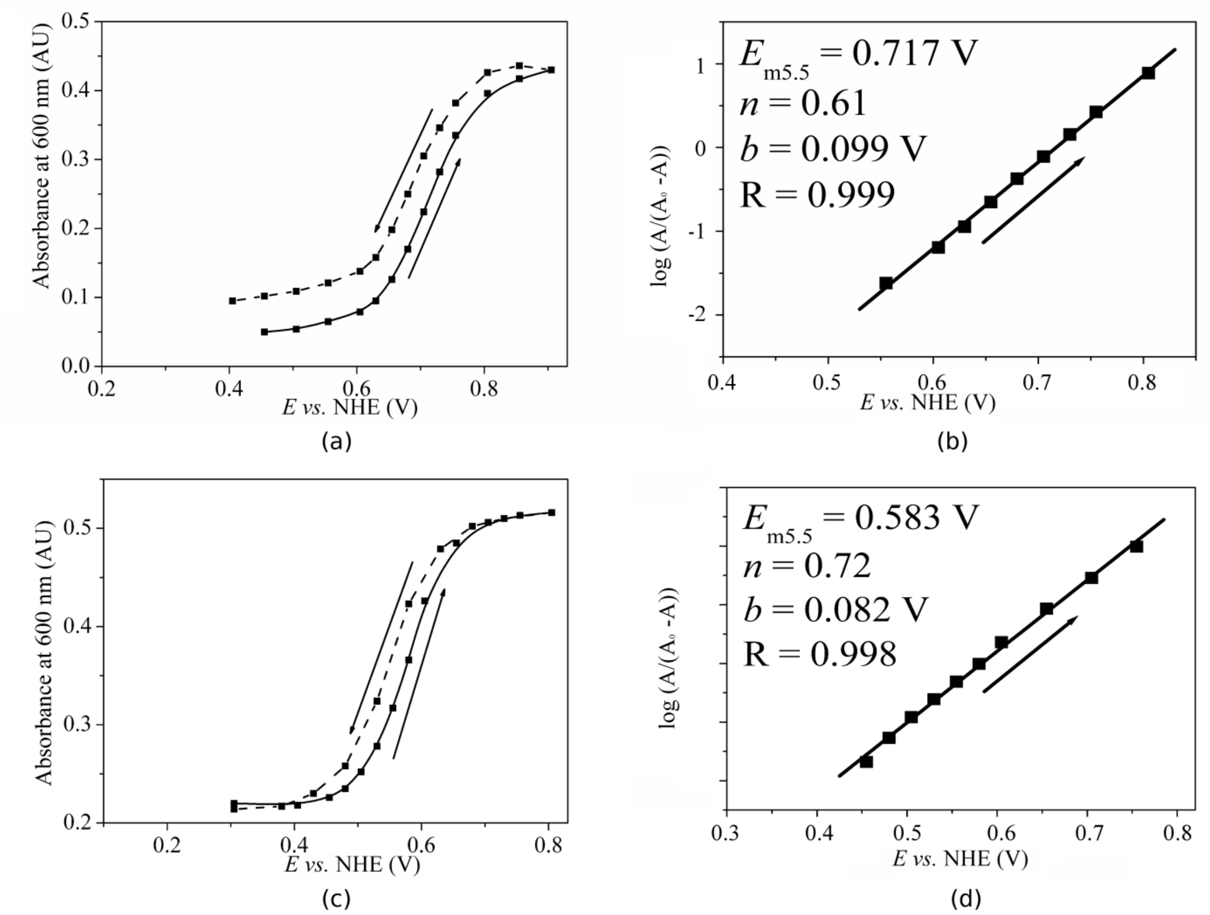

Figure S4 Mediated spectroelectrochemical redox titration of *Ba*L and L499M *Ba*L at pH 5.5: a,b) *Ba*L potentiometric titration curve and Nernst plot with averaged parameters calculated from the titration; c,d) L499M *Ba*L potentiometric titration curve and Nernst plot with averaged parameters calculated from the titration. E_m – midpoint potential, n – number of electrons, b – slope of the titration curve, R – correlation coefficient.

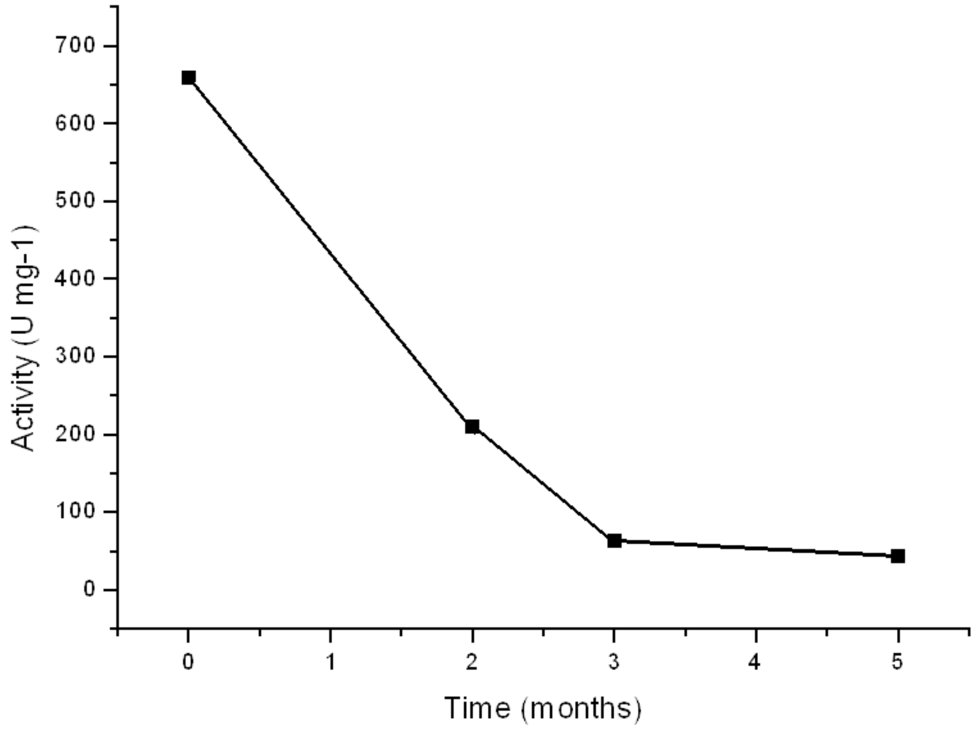

Figure S5 Loss of specific activity of deglycosylated BaL during storage in 0.1 M sodium citrate, pH 5.0, 0.5 M ammonium sulfate at +4 ^{0}C. The activity was measured at pH 4.0 using the ABTS as described in the Materials and Methods section
